# A comparison of methodological approaches to developing clinical prediction models for individuals living with multiple long-term conditions: a protocol for a systematic review

**DOI:** 10.1186/s41512-026-00221-2

**Published:** 2026-02-06

**Authors:** Lauren A Anderson, Joie Ensor, Clare L Gillies, Selina T Lock, Kamlesh Khunti, Laura J Gray

**Affiliations:** 1 Division of Public Health and Epidemiology, School of Medical Sciences, University of Leicester, Leicester, UK; 2 NIHR Leicester Biomedical Research Centre, University of Leicester, Leicester, UK; 3 Department of Applied Health Sciences, School of Health Sciences, College of Medicine and Health, University of Birmingham, Birmingham, UK; 4 National Institute for Health and Care Research (NIHR) Birmingham Biomedical Research Centre, Birmingham, UK; 5 Leicester Real World Evidence Unit, Diabetes Research Centre, Leicester, LE5 4PW UK; 6 Diabetes Research Centre, Leicester Diabetes Centre, Leicester General Hospital, Leicester, LE5 4PW UK; 7 Library and Learning Services, University of Leicester, Leicester, UK

**Keywords:** Systematic review, MLTCs, Prognostic modelling, Risk prediction, Clinical prediction models

## Abstract

**Introduction:**

Multiple long-term conditions (MLTCs) are being made a priority by funding bodies as prevalence rates increase. Improving early detection of individuals at high risk of developing MLTCs may delay or prevent complications and poor health outcomes. Predicting MLTCs remains a challenge, and methods for singular outcomes have been proven to be inappropriate for MLTC research. The aim of this paper is to present the protocol for a systematic review to identify all published models for prediction of MLTCs, and to summarise methods used for model development.

**Methods and analysis:**

MEDLINE (Ovid), Embase (Ovid), CINAHL (EBSCOHost) and CENTRAL (Cochrane Library) will be searched from September 2015 to identify relevant clinical prediction models which predict the development of MLTCs.

Screening, data extraction and the risk of bias will be undertaken by two reviewers independently. Data extraction will include primary items for methodology and model outcomes and secondary items including study descriptors, population information, measured outcomes, methodology, model performance measures, clinical usefulness measures and risk of bias. A narrative synthesis will be conducted to summarise current methodological practice and to identify areas for improvement to inform future methodological and model development.

**Ethics and dissemination:**

Ethical approval is not required for this systematic review as it will use published literature only. The findings of the review will be submitted for publication in a peer reviewed journal.

**Strengths and limitations of this study:**

• A comprehensive review of all methodologies used in clinical prediction modelling for MLTCs will be undertaken.

• Methodologies will be included regardless of the outcome conditions predicted.

• The protocol is reported according to the Preferred Reporting Items for Systematic Reviews Protocol (PRISMA – P).

• Screening, data extraction and risk of bias will be conducted independently by two reviewers.

• Only English language papers will be included.

**Supplementary Information:**

The online version contains supplementary material available at 10.1186/s41512-026-00221-2.

## Introduction

Multiple long-term conditions (MLTCs) refer to two or more co-occurring chronic conditions in an individual and is associated with an increased need for healthcare and a reduced quality of life [1]. Current prevalence estimates range from 0.9% for 0–19-year-olds to 68.2% in those 80 and over, with an average overall prevalence of 14.8% [2]. Odds of developing MLTCs are increased for deprived communities [2]. There is a move towards a more holistic approach to healthcare, as the prevalence of MLTCs rises, with funders, such as the National Institute of Health and Care Research (NIHR), making MLTCs a priority [3].

Despite the growing interest in MLTCs, research is limited by varying definitions of MLTCs and which conditions are incorporated [4]. This led to the development of an international consensus for the definition and measurement of MLTCs in research, which comprises of 24 conditions which should be included when researching MLTCs [5].

It is essential that risk factors for MLTCs are determined to better improve treatments and intervention strategies, as well as improve understanding of patterns of MLTCs and their burden [6]. Longitudinal studies are key here, as current research on the determinants of MLTCs has focused on cross-sectional studies, making it difficult to establish their impact [7]. For example some variables may be acting as a proxy for another factor, leading to various reasons why some associations have been observed [7].

However, prediction of MLTCs can be complicated. For example, some co-occurring conditions may have similar aetiology, and might require similar treatment options [7]. This is known as being concordant. However, those which seem unrelated to each other, or require different treatment are known as discordant [7]. Concordant conditions may cluster due to shared risk factors, but the latter may not appear to have any shared risk factors [8, 9]. Therefore, methodologies must be able to capture diverse outcomes.

Furthermore, interactions between factors is becoming increasingly recognised, and there are benefits to investigating how this produces condition clusters, such as improvements in the delivery of healthcare methods including integrating services and providing a more holistic approach to consider the impact of MLTCs on patients’ lives [7].

Currently treatment is focused around single conditions [10] and individuals with MLTCs have high levels of polypharmacy, which could lead to harm and the need to manage resulting effects [11]. Furthermore, treatments may need to be considered as confounders during risk prediction, to ensure accurate results [12].

Clinical prediction models (CPMs) also often consider a singular outcome, but in the case of MLTCs there is a need to estimate the risk of co-occurring conditions, which may require a multivariate approach [13]. One may be tempted to estimate the risk of multiple conditions by multiplying the risks from single-condition models. However, this operates under the assumption of independence, which is often violated in the case of MLTCs [14]. There is an absence of clear methodologies to handle this dependence between comorbidities [13] and the problem remains a challenge. This may be attributed to complexity in methodologies, yet it is clear bridging this gap is essential to improving risk estimation for MLTCs [14].

Methodologies for risk prediction for MLTCs is an emerging topic, yet there lacks a review in this area, which collates potential approaches and how and when to use them.

The aim of this paper is to present the protocol for a systematic review to identify all published models for the prediction of MLTCs, and to summarise methods used for model development. In doing so, it will identify areas that require further methodological development, and inform the methods for the development of a new model for MLTCs to be used in the UK.

## Methods and analysis

This protocol is reported according to the Preferred Reporting Items for Systematic Reviews Protocols (PRISMA-P) guidelines [15]. MLTCs can be defined as:

“The co-existence of two or more chronic conditions, each of which is either:

- A physical non-communicable disease of long duration, such as cardiovascular disease or cancer.
- A mental health condition of long duration, such as a mood disorder or dementia.
- An infectious disease of long duration, such as HIV or hepatitis C.” [7].

### Search

The following electronic databases will be used for searches: MEDLINE (Ovid), Embase (Ovid), CINAHL (EBSCOHost) and CENTRAL (Cochrane Library) to identify relevant clinical prediction models which predict the development of MLTCs. The start date will be 2015 and the end date will be the date on which the searches are completed. Screening will begin in September 2025 and results of the review are expected in early 2026.

A search strategy was developed using the Ingui filter for finding prognostic and diagnostic prediction studies [16], alongside a specialist librarian, to ensure optimal identification of relevant studies. The filter was adjusted when using Embase (Ovid), to ensure it was suitable. Search terms for MLTCs were found using research of the current literature. Both the prediction and MLTC terms were restricted to title and abstract searching, to ensure consistency across databases. A copy of the search strategies can be viewed in S1.

### Inclusion criteria

- Population: Adults (aged 18 and over). Only studies including adults will be eligible for this review. Populations can be disease free or have a single long-term condition at baseline.
- Reporting of a CPM which has been developed to predict the risk of developing more than one new long-term condition. A CPM will be defined as a model incorporating at least two predictors, providing a probability or risk of a future outcome of MLTCs.
- Time horizon: Publications from 2015 onwards to focus on contemporary models, ensuring the findings reflect the current field.
- Setting: Any setting or context.
- Peer reviewed articles.

Only full text articles, available in English will be included. This restriction is due to the methodological nature of this review, which requires an excellent comprehension of the technicalities of the methods. The number of papers excluded due to language restraints will be reported.

### Exclusion criteria

Studies that do not involve the development of a CPM, for more than one long-term condition, will not be included. Studies only available in abstract form will be excluded, including conference abstracts and pre-prints. Protocols, systematic reviews and grey literature will also be excluded.

### Study selection

All eligible studies from the literature search will be imported into Covidence and duplicates will be removed. Titles and abstracts will be screened first by two independent reviewers, following the inclusion and exclusion criteria. Conflicts will be resolved by discussion with a third reviewer. There will be further screening on the full text of articles, conducted by two independent reviewers, with conflicts resolved by a third. Papers that are excluded will have the reason for exclusion recorded.

### Data extraction

All data will be extracted independently by two reviewers, with disagreements discussed with a third. The CHARMS checklist has informed the data extraction [17]. This will include:

Primary outcomes related to the methodological approach and any restriction upon using the method, including:

- Methods: methodology used to develop CPM, any alternative presentation of the model.
- Outcomes: timespan of prediction/follow-up years, format of outcome (e.g. binary, continuous, categorical) and whether outcomes were transformed to an alternative format, number of outcomes.

Secondary outcomes:

- Study descriptors: Title, authors, study dates, publication date, journal, country, setting, number of patients.
- Population: inclusion/exclusion criteria, sample size, age range, baseline condition status, number of participants with missing data.
- Outcomes measured: type of long-term conditions,
- Methods used: handling of missing data, which candidate predictors were used and any combining of predictors, structure of predictors included, consideration of proxies.
- Model performance measures: calibration, discrimination and overall performance statistics, internal and external validation.
- Clinical usefulness measures such as net benefit, and consideration of impact on health outcomes.
- Risk of bias: Risk of bias will be assessed using the PROBAST + AI tool [18] with two independent reviewers assessing included studies in regard to participants, predictors, outcome and analysis. Studies will then be rated as low, high or unclear risk of bias.

Primary outcomes being prioritised are those related to the main aim of this study, providing information regarding the methodological approaches used in the literature, and when they are appropriate to be implemented, including any restrictions on their use. Secondary outcomes will help with study identification, as well as providing additional information indicating the quality of the study.

### Data synthesis

A narrative synthesis will be conducted to summarise the main characteristics of the papers, including the methodologies used, and key aspects of model development. These will be further presented with tables and figures, including the risk of bias (PROBAST + AI). Methodologies will be compared and grouped by outcome type, and any restrictions placed upon use, such as number of outcome conditions. Recommendations will be conducted in relation to application/restrictions as well as ease of use and risk of bias.

### Ethics and dissemination

Ethical approval is not required for this systematic review as it will use published literature only. The findings of the review will be submitted for publication in a peer reviewed journal.

## Discussion

Prevalence of MLTCs is rising and thus there is an increased need for methodologies which aid risk prediction, leading to early intervention and improved MLTC management, ultimately aiming to reduce the burden of MLTCs and the impact of subsequent complications. However, research on methodology for MLTCs is still in its early stages and presents with various challenges. This is enhanced by the varying aetiology of conditions, and the vast number of combinations of conditions which exist. Nevertheless, bridging this gap in the research is essential to improving outcomes for individuals with MLTCs, as it may aid in improving identification of those living with MLTCs, leading to better condition management and treatment.

This systematic review addresses this need by synthesising existing prognostic model studies in this area, providing a comprehensive overview of the existing literature. Furthermore, it will provide insights into the methodological challenges faced in this area.

The systematic review is strengthened by its focus on performance metrics, which will allow comparison of the applicability of model methods. Furthermore, the use of the CHARMs checklist and PROBAST + AI risk of bias tool will ensure a comprehensive assessment of study quality and methodology robustness. However, the review is subject to some limitations, such as the restriction to English-language papers, which may introduce bias.

Overall, once completed, the review could help inform future model development in this field and highlight methodological strengths and limitations. By collating the literature, it may assist in easy identification of existing methodologies for MLTC prediction, which could be useful for those conducting research in this area. Furthermore, providing information on their specific applications and restrictions, including strengths and limitations of methodologies, will allow researchers to make an informed choice regarding which method is appropriate for their research question.

## Conclusion

To our knowledge this systematic review will be the first to comprehensively synthesise the existing literature on methodologies for the development of CPMs for MLTCs. By identifying the strengths and limitations, and comparing by outcome and restrictions placed upon use, the review will help better inform current practices and improve model development in this area. Subsequently, this could provide better health outcomes for individuals with MLTCs, by improving early detection and condition management.

## Supplementary Information

Supplementary Material 1: S1 File. Search Strategies.

Supplementary Material 2: S2 File. PRISMA-P Checklist.

## Data Availability

No datasets were generated or analysed during the current study.

## Abbreviations

MLTCs: Multiple Long–Term Conditions
CPMs: Clinical Prediction Models
