# Supplementary material for: A comparison of methodological approaches to developing clinical prediction models for individuals living with multiple long-term conditions: a protocol for a systematic review
Source: Diagn Progn Res. 2026 Feb 6;10:6. doi: 10.1186/s41512-026-00221-2 (PMC12879393; doi:10.1186/s41512-026-00221-2)
Supplement: Supplementary file 1 — Supplementary Material 1: S1 File. Search Strategies. [file 41512_2026_221_MOESM1_ESM.docx]

**Search strategy MEDLINE (Ovid)**

1 exp Multimorbidity/

2 (multimorbid* or multi-morbid*).ti,ab.

3 (multidisease* or multi-disease* or multicondition* or polymorbid* or poly-morbid*).ti,ab.

4 (multiple chronic condition* or multiple chronic disease* or multiple chronic ill*).ti,ab.

5 (multiple long-term condition* or multiple long term condition*).ti,ab.

6 ((multiple or multi) adj (morbid* or ill* or disease* or condition* or syndrom* or disorder* or health or chronic)).ti,ab.

7 (multi-condition or MLTC).ti,ab.

8 exp Syndemic/

9 syndemic.ti,ab.

10 (multiple long-term health condition* or multiple long term health condition*).ti,ab.

11 ((co-occurring or coocurring) adj (health or condition*)).ti,ab.

12 (cluster* adj (health or condition*)).ti,ab.

13 (concurren* adj (illness* or disease* or disorder* or condition*)).ti,ab.

14 (polypatholog* or poly-patholog*).ti,ab.

15 (pluripatholog* or pluri-patholog*).ti,ab.

16 (multipatholog* or multi-patholog*).ti,ab.

17 1 or 2 or 3 or 4 or 5 or 6 or 7 or 8 or 9 or 10 or 11 or 12 or 13 or 14 or 15 or 16

18 Validat*.ti,ab. or Predict*.ti. or Rule*.ti,ab.

19 (Predict* and (Outcome* or Risk* or Model*)).ti,ab.

20 ((History or Variable* or Criteria or Scor* or Characteristic* or Finding* or Factor*) and (Predict* or Model* or Decision* or Identif* or Prognos*)).ti,ab.

21 (Decision* and (Model* or Clinical*)).ti,ab.

22 Decision*.ti,ab. and Logistic Models/

23 (Prognostic and (History or Variable* or Criteria or Scor* or Characteristic* or Finding* or Factor* or Model*)).ti,ab.

24 18 or 19 or 20 or 21 or 22 or 23

25 17 and 24

26 limit 25 to (english language and yr="2015 -Current")

**Search strategy CINAHL (EBSCOHost)**

S1 XB (multimorbid* OR multi-morbid*)

S2 XB (multidisease* OR multi-disease* OR multicondition* OR polymorbid* OR poly-morbid*)

S3 XB ("multiple chronic condition*" OR "multiple chronic disease*" OR "multiple chronic ill*))

S4 XB ("multiple long-term condition*" OR "multiple long term condition*")

S5 XB ((multiple OR multi) W1 (morbid* OR ill* OR disease* OR condition* OR syndrom* OR disorder* OR health OR chronic))

S6 XB ((multi-condition OR MLTC))

S7 (MH "Syndemic")

S8 XB syndemic

S9 XB ("multiple long-term health condition*" OR "multiple long term health condition*")

S10 XB ((co-occurring OR cooccurring) W1 (health OR condition*))

S11 XB (cluster* W1 (health OR condition*))

S12 XB (concurren* W1 (illness* OR disease* OR disorder* OR condition*))

S13 XB (polypatholog* OR poly-patholog*)

S14 XB (pluripatholog* OR pluri-patholog*)

S15 XB (multipatholog* OR multi-patholog*)

S16 S1 OR S2 OR S3 OR S4 OR S5 OR S6 OR S7 OR S8 OR S9 OR S10 OR S11 OR S12 OR S13 OR S14 OR S15

S17 XB Validat* OR TI Predict* OR XB Rule*

S18 XB (Predict* AND (Outcome* OR Risk* OR Model*))

S19 XB ((History OR Variable* OR Criteria OR Scor* OR Characteristic* OR Finding* OR Factor*) AND (Predict* OR Model* OR Decision* OR Identif* OR Prognos*)))

S20 XB (Decision* AND (Model* OR Clinical*))

S21 XB (Decision* AND "Logistic Models")

S22 XB ((Prognostic AND (History OR Variable* OR Criteria OR Scor* OR Characteristic* OR Finding* OR Factor* OR Model*)))

S23 S17 OR S18 OR S19 OR S20 OR S21 OR S22

S24 S16 AND S23  **Limiters** - Publication Date: 20150101-20251231

**Expanders** - Apply equivalent subjects

**Narrow by Language:**- english

**Search modes** – Proximity

**Search strategy CENTRAL (Cochrane Library)**

MeSH descriptor: [Multimorbidity] explode all trees

#2 multimorbid*:ti,ab OR multi-morbid*:ti,ab

#3 multidisease*:ti,ab OR multi-disease*:ti,ab OR multicondition*:ti,ab OR polymorbid*:ti,ab OR poly-morbid*:ti,ab

#4 (("multiple chronic" NEXT condition*):ti,ab OR ("multiple chronic" NEXT disease*):ti,ab OR ("multiple chronic" NEXT ill*):ti,ab)

#5 (("multiple long-term" NEXT condition*):ti,ab OR ("multiple long term" NEXT condition*):ti,ab)

#6 (multiple OR multi):ti,ab NEXT (morbid* OR ill* OR disease* OR condition* OR syndrom* OR disorder* OR health OR chronic):ti,ab

#7 multi-condition:ti,ab OR MLTC:ti,ab

#8 MeSH descriptor: [Syndemic] explode all trees

#9 syndemic:ti,ab

#10 (“multiple long-term health" NEXT condition*):ti,ab OR (“multiple long term health" NEXT condition*):ti,ab

#11 (co-occurring OR cooccurring):ti,ab NEXT (health OR condition*):ti,ab

#12 cluster*:ti,ab NEXT (health OR condition*):ti,ab

#13 concurren*:ti,ab NEXT (illness* OR disease* OR disorder* OR condition*):ti,ab

#14 polypatholog*:ti,ab OR poly-patholog*:ti,ab

#15 pluripatholog*:ti,ab OR pluripatholog*:ti,ab

#16 multipatholog*:ti,ab OR multi-patholog*:ti,ab

#17 #1 OR #2 OR #3 OR #4 OR #5 OR #6 OR #7 OR #8 OR #9 OR #10 OR #11 OR #12 OR #13 OR #14 OR #15 OR #16

#18 validat*:ti,ab OR predict*:ti OR rule*:ti,ab

#19 predict*:ti,ab AND (outcome* OR risk* OR model*):ti,ab

#20 (history OR variable* OR criteria OR scor* OR characteristic* OR finding* OR factor*):ti,ab AND (predict* OR model* OR decision* OR identif* OR prognos*):ti,ab

#21 decision*:ti,ab AND (model* OR clinical*):ti,ab

#22 (decision* AND "logistic models"):ti,ab

#23 prognostic:ti,ab AND (history OR variable* OR criteria OR scor* OR characteristic* OR finding* OR factor* OR model*):ti,ab

#24 #18 OR #19 OR #20 OR #21 OR #22 OR #23

#25 #17 AND #24 with Publication Year from 2015 to present, in Trials

**Search strategy Embase (Ovid)**

1 exp multiple chronic conditions/

2 (multimorbid* or multi-morbid*).ti,ab.

3 (multidisease* or multi-disease* or multicondition* or polymorbid* or poly-morbid*).ti,ab.

4 (multiple chronic condition* or multiple chronic disease* or multiple chronic ill*).ti,ab.

5 (multiple long-term condition* or multiple long term condition*).ti,ab.

6 ((multiple or multi) adj (Morbid* or ill* or disease* or condition* or syndrom* or disorder* or health or chronic)).ti,ab.

7 (multi-condition or MLTC).ti,ab.

8 exp syndemic/

9 syndemic.ti,ab.

10 (multiple long-term health condition* or multiple long term health condition*).ti,ab.

11 ((co-occurring or coocurring) adj (health or condition*)).ti,ab.

12 (cluster* adj (health or condition*)).ti,ab.

13 (concurren* adj (illness* or disease* or disorder* or condition*)).ti,ab.

14 (polypatholog* or poly-patholog*).ti,ab.

15 (pluripatholog* or pluri-patholog*).ti,ab.

16 (multipatholog* or multi-patholog*).ti,ab.

17 1 or 2 or 3 or 4 or 5 or 6 or 7 or 8 or 9 or 10 or 11 or 12 or 13 or 14 or 15 or 16

18 Validat*.ti,ab. or Predict*.ti. or Rule*.ti,ab.

19 (Predict* and (Outcome* or Risk* or Model*)).ti,ab.

20 ((History or Variable* or Criteria or Scor* or Characteristic* or Finding* or Factor*) and (Predict* or Model* or Decision* or Identif* or Prognos*)).ti,ab.

21 (Decision* and (Model* or Clinical*)).ti,ab.

22 Decision*.ti,ab. and statistical model/

23 (Prognostic and (History or Variable* or Criteria or Scor* or Characteristic* or Finding* or Factor* or Model*)).ti,ab.

24 18 or 19 or 20 or 21 or 22 or 23

25 17 and 24

26 limit 25 to (english language and yr="2015 -Current")
